# Readiness for interprofessional learning among health science students: a cross-sectional Q-methodology and likert-based study

**DOI:** 10.1186/s12909-023-04566-w

**Published:** 2023-08-18

**Authors:** Ana Oliveira, Danielle Brewer-Deluce, Noori Akhtar-Danesh, Sarah Wojkowski

**Affiliations:** 1 Program for Interprofessional Practice, Education and Research (PIPER), McMaster University, 1400 Main Street West, Hamilton, ON L8S 1C7, 647-765- 1525 Canada; 2 Respiratory Medicine, West Park Healthcare Centre, Toronto, Canada; 3 Lab3R – Respiratory Research and Rehabilitation Laboratory, School of Health Sciences, University of Aveiro (ESSUA), Aveiro, Portugal; 4 Department of Medical Sciences, iBiMED – Institute of Biomedicine, University of Aveiro, Aveiro, Portugal; 5 Faculty of Health Sciences, McMaster University, Hamilton, Canada

**Keywords:** Q-methods, RIPLS, Learners, Interprofessional collaboration

## Abstract

**Background:**

Interprofessional education (IPE) prepares healthcare students for collaboration in clinical practice, but the effectiveness of this teaching method depends on students’ readiness for and perceptions of IPE. Evaluating students’ readiness for and perceptions of IPE is challenging, due to the lack of comprehensive measures. This study characterized the level of IPE readiness and perspectives across first-year undergraduate and graduate health science students using the readiness for interprofessional learning Likert Scale (RIPLS) and Q-methodologies.

**Methods:**

This is a cross-sectional, online study. Students were randomized to answer the Likert-scale version of RIPLS (80%) or a matched Q-methodology survey (20%). An ANCOVA compared RIPLS scores between students from different program levels (graduate/undergraduate) and specialization (health professional and general programs). The Q-data was analysed using a by-person factor analysis.

**Results:**

Three hundred and four (33% response rate) and 71 (30% response rate) students completed the Likert scale and the Q-methodology surveys, respectively. Students from graduate programs demonstrated high readiness for IPE (higher total RIPLS scores p < 0.001) in comparison to undergraduates. Three factors, associated with program specialization (p = 0.04), emerged from the Q-methodology analysis characterizing students learning priorities. Students in undergraduate general programs were focused on IPE relevance and benefits to “the clinical team”, students in graduate programs focused on “the patient”, and those in undergraduate health professional programs focused on themselves (“me”).

**Conclusions:**

This novel mixed-methods approach combining traditional Likert-scales with Q-methodology elucidated not only associations between program and specialization with readiness (Likert) but also which components of IPE were valued the most (Q-methodology) and by whom.

**Supplementary Information:**

The online version contains supplementary material available at 10.1186/s12909-023-04566-w.

## Introduction

Interprofessional collaboration (IPC) is a key strategy for healthcare reform [1] as it improves patients’ outcomes (e.g., reducing adverse drug reactions, morbidity and mortality rates) and healthcare providers’ satisfaction (e.g., reducing extra work and increasing job satisfaction) [2]. Thus, it has become an expectation of health professional preparation that students will be ready for, and capable of, effective interprofessional work at graduation [3].

Interprofessional education (IPE) is an experience that “occurs when students from two or more professions learn about, from, and with each other” [4] and, when introduced at early training stages, has been shown to be effective in preparing students for IPC in clinical practice, by improving the collaborative team behavior and reducing clinical error [5, 6]. For IPE to be a positive experience, students must be willing and ready to engage in cooperative learning with others [7, 8]. Thus, understanding the attitudes and perceptions of students before they encounter IPE events is a critical first step for the development and implementation of stage-matched educational interventions and for the effectiveness of such events [9].

## Background

The complexity of healthcare globally has been a driving force for the implementation of IPE [4]. In Canada, experience with IPE in health science curricula is a requirement of most accreditation bodies [10–12]. Nevertheless, the introduction of IPE into professional curriculum is complex and requires a thorough evaluation - not only to guide educators concerning the quality of learning [13], but also to ensure students are ready for collaborative practice.

The Readiness for Interprofessional Learning Scale (RIPLS) is a self-report scale that allows for the evaluation of students’ readiness for interprofessional learning [14]. Originating in 1999, the scale has been widely used within the IPE community primarily due to its ease of administration, potential for establishing comparisons among individuals and populations, and validity across multiple professional disciplines (e.g., medicine, dentistry, physiotherapy, nursing, occupational therapy, orthopedics, therapy radiography and diagnostic radiography) [14, 15]. However, like most scales in the education field [16–19], the RIPLS is a Likert scale questionnaire, which has intrinsic limitations in assessing attitudes and perceptions [20]. An important limitation of the RIPLS is that it provides numerical results for psychological constructs that intrinsically lack quantitative structure [20]. Further, numerical rankings do not easily translate into a meaningful representation of the student experience [21] as the diversity of perceptions across individuals and groups are obscured by the calculation of a mean and standard deviation. This may be of particular importance for healthcare educators who aim to better understand students’ perceptions of IPE and adjust IPE curricula towards student needs and level of readiness.

An alternate methodology available to capture the uniqueness and diversity in a groups’ perspective is Q-methodology. Q-methodology is used to understand patterns of thought within a given sample [22] and is based on the notion that subjectivity is both communicable and self-referent [20]. Unlike the normative approach used in Likert-type surveys, Q-methodology allows students to assess each item in an ipsative manner (i.e., participants assign a psychological response to item based on the item’s relative ranking compared with all other items) [20] and provide critical reasoning for their choices [23]. Q-methodology is, however, not without limitations; and an important one is that the method is time consuming, as Q-methodology requires significantly more expertise and dedicated time to create, deliver and analyze in comparison to Likert scales.

With this in mind, and while acknowledging the novelty of this approach, we posit that by combining the numeric and generalizable strengths of Likert scales, with the subjective and psychologically grounded perspectives emerging from Q-methodology, we will gain critical complementary information about students’ readiness for IPE (“how much”) and their unique vision about IPE (“how diverse”). Ultimately this stands to provide a more rounded and accurate understanding of the students’ perspective about IPE to inform research and educational decision-making.

The purpose of this study was to characterize the level of IPE readiness and perspectives across incoming health science students using the RIPLS scale and a matched Q-methodology survey, in which the Q-sorts were derived from statements in the RIPLS scale.

## Materials and methods

### Ethical considerations

Students were informed that the survey was optional and anonymous, and that they would provide consent for their information to be included in the study by submitting the questionnaires. No incentives were offered for participation. To ensure student anonymity, each student was responsible for creating their own unique ID number following a series of instructions (e.g., first Initial, last 3 letters from last name, birth month short form). Only composite data, without associated IDs, was shared with Program heads/chairs as an additional step to guarantee anonymity of all responses. The study protocol and aim underwent evaluation by the Hamilton Integrated Research Ethics Board, and a letter of exemption from requiring ethics approval was provided, considering the study’s classification as a quality assurance initiative.

### Research design and data collection

This cross-sectional study included first-year health science students from five undergraduate health professional programs (HPP) four graduate HPP and one undergraduate general program of the Faculty of Health Sciences at McMaster University (Canada). There were no graduate general programs offered and thus available for inclusion in the study (Table 1).

**Table 1:** Health professional programs

	Graduate	Undergraduate
Health Professional Program	Physiotherapy Occupational Therapy Child Life Speech Language Pathology	Medicine Nursing Midwifery Physician’s Assistant Social Work
General	-	Bachelor of Health Sciences

Four weeks before initiating their respective programs, 1158 potential participants were invited to answer one of two online versions of the RIPLS: the traditional Likert scale by McFadyen et al. (2005) [24] using an online learning management system (Avenue to Learn) and the Q-methodology survey using a specifically designed electronic platform (a demonstration version can be found at https://macanatomy.mcmaster.ca/q/qdemo/#/). Data from the Q-methodology survey were analyzed via by-person factor analysis [25] and interpretation of qualitative feedback. For each version of the RIPLS, participants were provided with a brief explanation of the scale and instructions for its completion.

To reduce the burden of research in students, participants were randomized so that 80% would be offered the Likert-scale version of the RIPLS (n = 922 from which 304 responded) and 20% were offered the Q-methodology survey version (n = 236, from which 71 responded). Since the goal of Q-methodology is to identify typologies within a cohort, low response rates do not bias results [26].

**Fig. 1:**
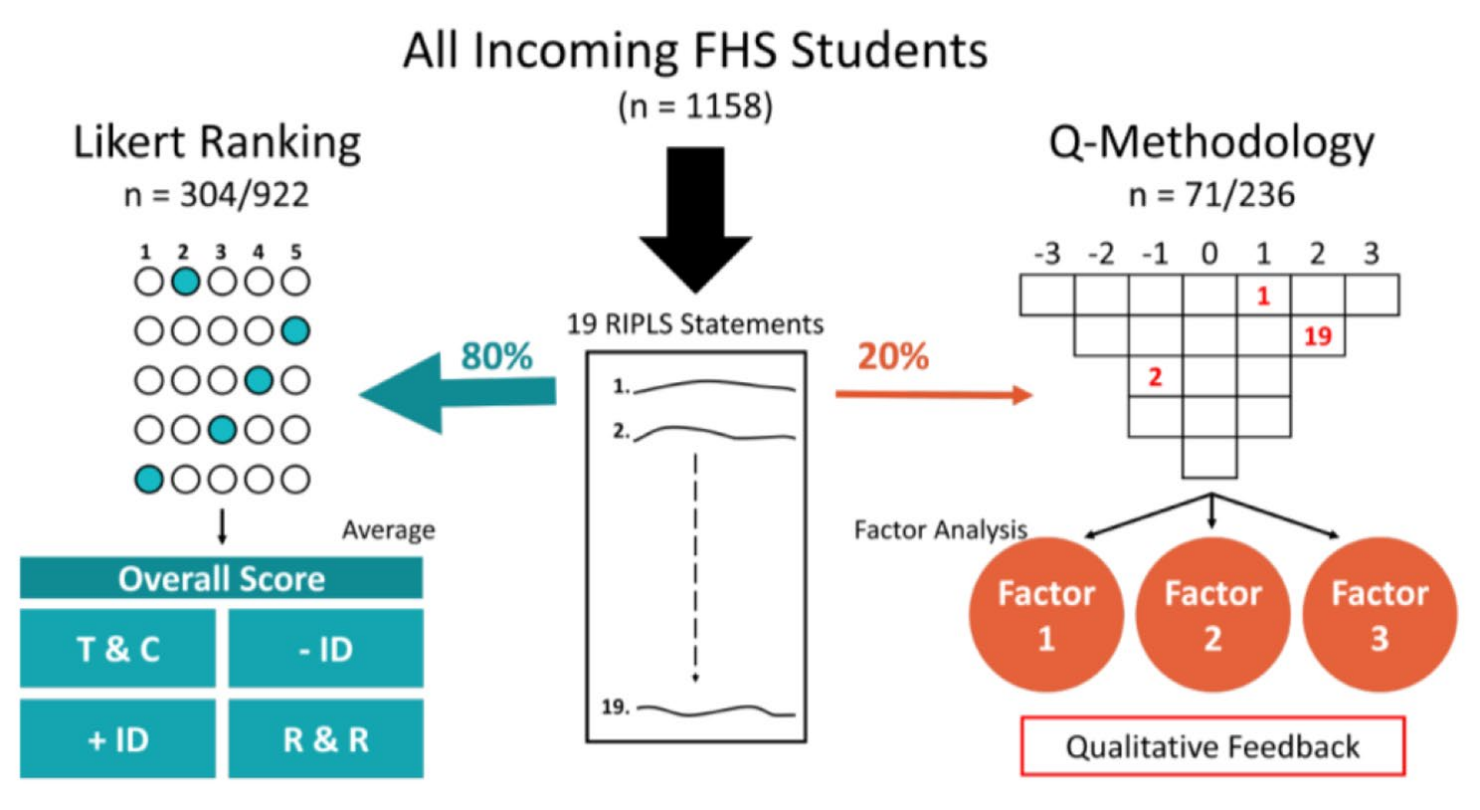
Study organization: 1158 students were invited to participate in this study and were randomly assigned to complete the traditional Likert-Ranking (80% of population) or a Q-methodology ranking of the 19 RIPLS statements (20% of population). Likert rankings (n = 304/922) were summed to create an overall score as well as the 4 subscales as described by McFadyen et al. (2005). Q-methodology rankings (n = 71/236) were analyzed via by-person factor analysis and interpretation of qualitative feedback. Legend: T&C: Teamwork & collaboration; -ID, Negative professional identity; +ID, positive professional identity; R&R, roles and responsibilities Legend: T&C: Teamwork & collaboration; -ID, Negative professional identity; +ID, positive professional identity; R&R, roles and responsibilities

A graphical representation of data collection of the two RIPLS versions is in Fig. 1.

Sociodemographic (year of birth, gender, and health science program) and IPE-related variables (previous experiences with IPE) were also collected.

### Outcome measures

#### Likert-scale version of the RIPLS

Readiness for interprofessional learning was measured with the a Likert-scale version of the RIPLS questionnaire previously validated by McFadyen et al. [24]. This version of the scale was chosen as it has proved to be reliable and more stable than previous published versions [24]. The scale consists of 19 statements (online supplement 1) for which students rank their personal agreement using a 5-point Likert scale (1 = strongly disagree, 5 = strongly agree) [24]. Importantly, negative statements (items 10–12, 17–19) are scored in reverse such that a higher overall score indicates a higher readiness for interprofessional learning [24]. The scale can be interpreted as a whole score (average of the sum of Likert rankings across all 19 items) and in four subscales: teamwork and collaboration (items 1–9), negative professional identity (items 10–12), positive professional identity (items 13–16) and roles and responsibility (items 17–19). The total average score of this scale ranges from 0 to 5 with higher scores indicating greater readiness for IPE.

#### Q-methodology RIPLS

Q-methodology allows grouping people based on the similarities of their statement rankings via by-person factor analysis. By convention, Q study statements span positive, negative and neutral opinions about the topic of study and are ranked, by participants, relative to each other [22].

For this study, the 19 statements of the RIPLS were used as the Q-sample. A Q-sort table was then developed with 19 cells, so that each statement could be ranked and ordered within the table to permit subsequent analyses. The Q-sort table approximates a normal distribution, such that the statement ranking assumes a forced normal distribution between strongly agree (+ 3) and strongly disagree (-3).

To complete the study, participants were provided with the 19 RIPLS statements and a Q-sort table via a webpage. Following the methodology outlined by Brewer-Deluce, Sharma [23], participants were instructed to read the Q-statements carefully and rank them coarsely into “disagree”, “neutral” and “agree” categories. Then, participants would further specify the specific ranking they wished to associate with each statement by assigning it to an available cell in the Q-sort table. Statements could be rearranged by dragging and dropping the statement to a new cell until students were happy with their final sort, which they then submitted.

**Fig. 2:**
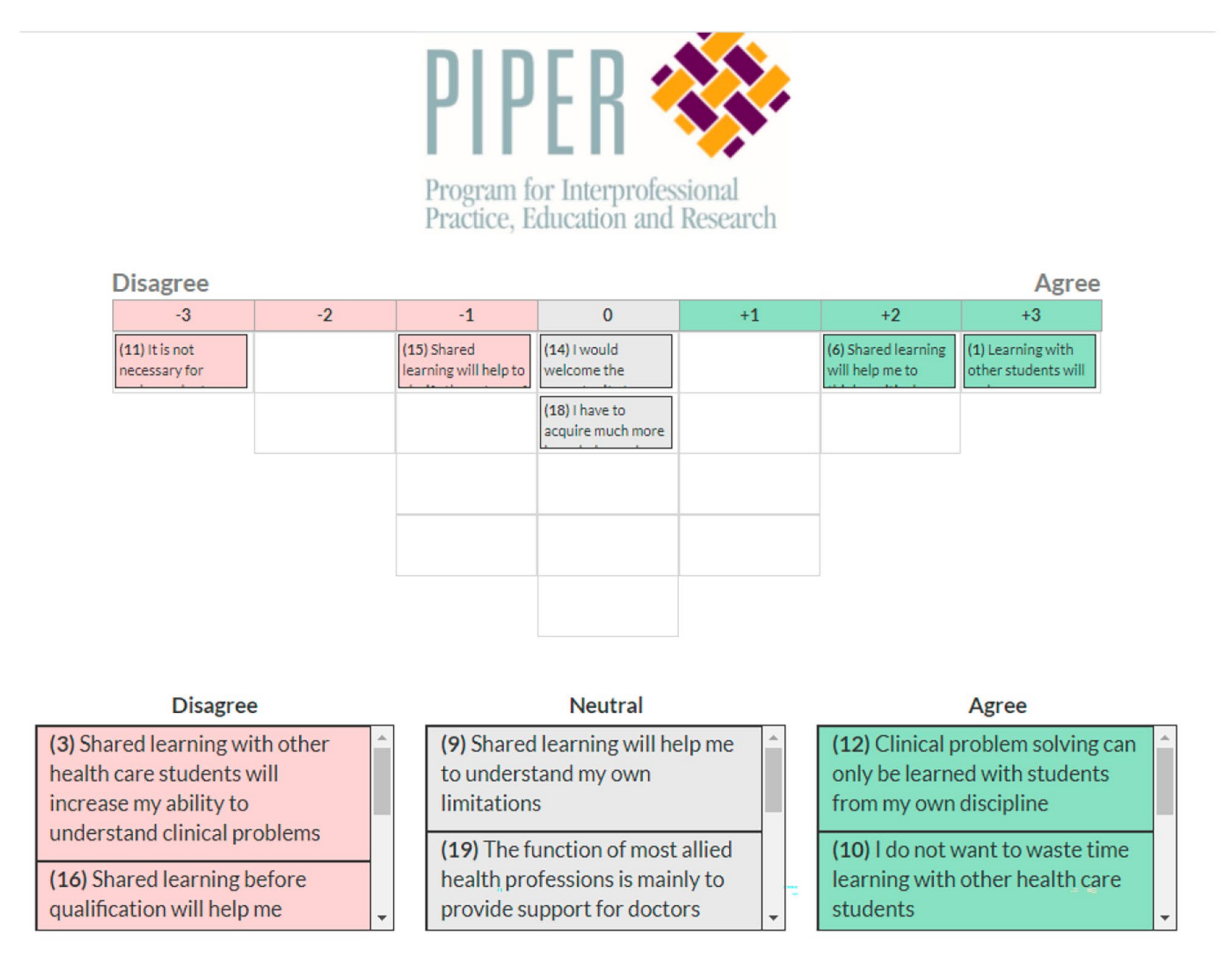
Screenshot of the Q-scoring system presented to participants. After initially coarsely sorting the Q-statements into “disagree”, “neutral” and “agree” categories, participants would further specify the specific ranking they wished to associate with each statement by assigning it to an available cell in the Q-sort table. Statements could be rearranged at will by dragging and dropping the statement to a new cell until students were happy with their final sort, which they then submitted

Statements ranked under the “0” (zero) column reflect neither agreement nor disagreement. Each cell in the Q-sort table needed to be filled, and only one statement could be assigned. In cases where there were multiple cells for a given ranking (e.g., two statements could be ranked − 2), participants were informed the order in which they place two statements did not need to be considered. Finally, for the responses at either extreme (+ 3 and − 3, termed critical statements), participants were asked to write a brief statement to contextualize or justify their response. The completed data set of sorted statements constitutes the Q-sort. A representation of the digital Q-sort system is in Fig. 2.

### Data analysis

#### Likert scale

Statistical analysis was carried out using SPSS (Statistical Package for the Social Sciences, Version 26.0; SPSS Inc., Chicago, IL, USA).

Descriptive statistics were used to characterise the sample and are presented as frequencies, percentages, means, and standard deviations. Baseline characteristics of the participants answering the Likert scale and the Q-methodology RIPLS were compared using independent t-tests. A two-way analysis of covariance (ANCOVA) was used to compare RIPLS scores between students from different program levels (i.e., graduate and undergraduate students) and level of program specialization (HPP and general programs), adjusting for age and gender (i.e., male, female, other, prefer not to say).

#### Q-methodology

Raw data were imported into Stata and a by-person factor analysis was performed using the “qfactor” command [25] to identify factors (i.e., groups of “individuals with similar views, feelings or experiences”) in the sample. Q-Factor scores for each statement were then calculated as a weighted average, and compared between factors [27]. Those which statistically significantly differ between factors are termed “distinguishing statements”, while those which do not statistically significantly differ between any factors are termed “consensus statements”. A Cohen’s effect size of 0.80 was used to identify distinguishing statements [25].

Qualitative data from the Q-sort pertaining to critical statements were interpreted concomitantly by the study team to generate more intuitive group names. Finally, the characteristics of students in each factor were analysed using Chi-square statistics to verify if the different factors identified were consistent with different student groups (i.e., undergraduates HPP; graduates HPP and undergraduates general).

## Results

### Sample characteristics

In total, 1158 first year students in health-related undergraduate and graduate programs were invited to participate by answering either the Likert scale (n = 922) or the Q-methodology survey (n = 236). From these, 304 (33% response rate) and 71 (30% response rate) completed the Likert scale or the Q- methodology survey, respectively. Response rate by program is in online supplement 2.

Participants had a mean age of 21.4 (SD = 4.3) years and were mainly women (n = 140; 37.3%) from undergraduate programs (n = 227; 60.5%) with no previous experience in IPE (n = 362; 96.5%). Differences between the demographic characteristics of students answering the Likert scale and the Q-methods RIPLS were only observed for gender (Table 2).

**Table 2:** Sample characteristics

	Likert scale RIPLS (n = 304)	Q- methods RIPLS (n = 71)	p-value
Age, years (median, [Q1-Q3])	22 [18–23]	22 [17–46]	0.467
Gender (n,%)			< 0.001*
Females	80 (26.3)	60 (84.5)	
Males	78 (25.7)	8 (11.3)	
Other	66 (21.7)	0 (0.0)	
Not reported	80 (26.3)	3 (4.2)	
Program level (n,%)			0.343
Undergraduate	186 (61)	41 (58)	
Graduate	118 (39)	30 (42)	
Program specialization (n,%)			0.554
Undergraduate – Health professional programs	98 (32.2)	25 (35)	
Undergraduate – General	88 (29.0)	16 (23)	
Graduate – Health professional programs	118 (38.8)	30 (42)	
No previous IPE (n,%)	291 (96)	71 (100)	0.202
Response rate, %	33	30	-

**Legend:** IPE, interprofessional education; RIPLS, readiness for interprofessional learning scale. *Statistical significance for p < 0.05

### Likert scale results

**Fig. 3:**
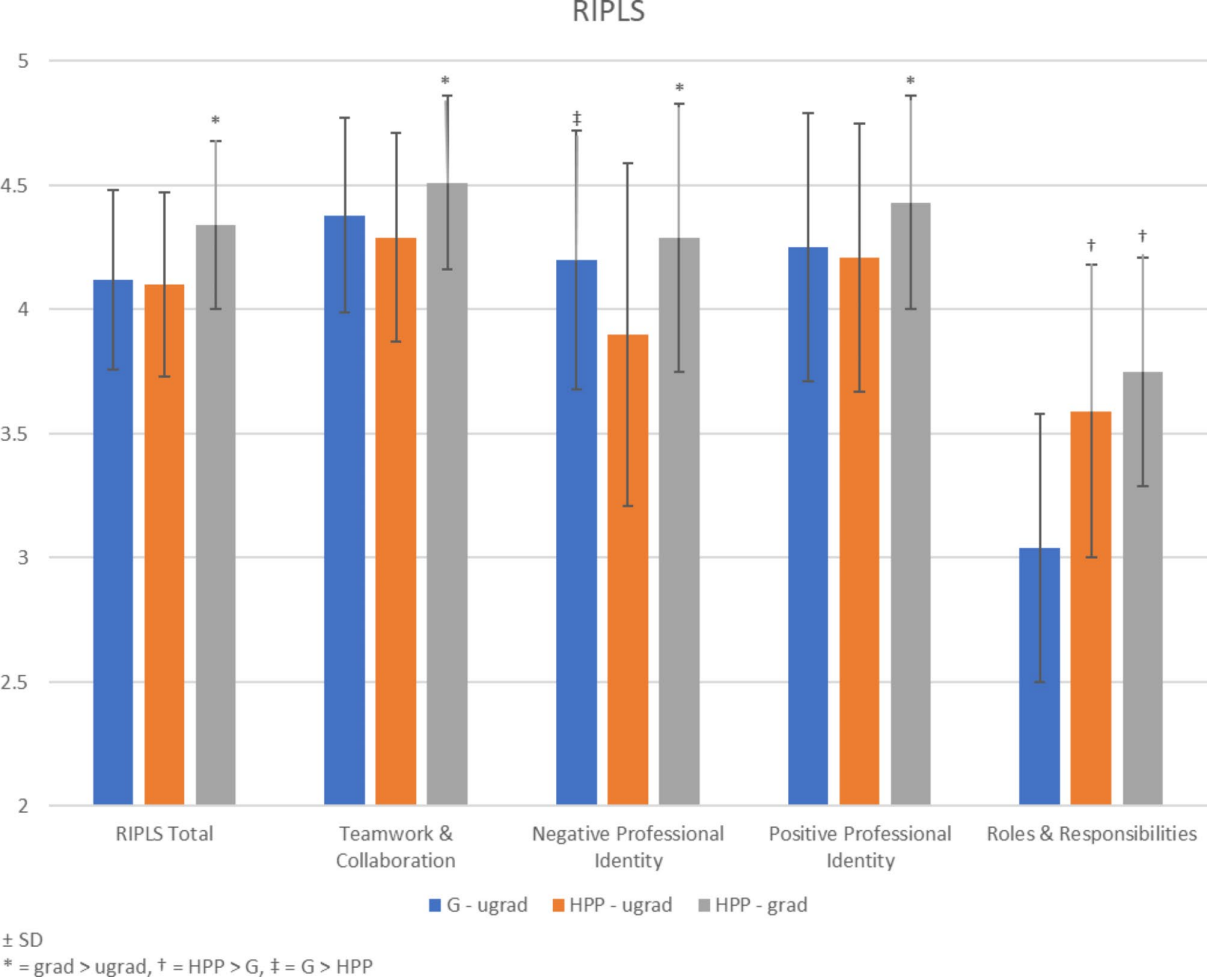
Scores of the Readiness for Interprofessional Learning Scale in participants from general (G), healthcare professional (HPP), undergraduate (ugrad), and graduate programs (grad) (controlled for age and gender)

A significant main effect of program level (F(1,298) = 25.771, p < 0.001) was found where graduate students’ total RIPLS scores exceeded those from undergraduates. There was no effect of program specialization or interaction. The same pattern held for the teamwork & collaboration (F(1,298) = 20.757, p < 0.001) and positive professional identity subscales (F(1,298) = 11.876, p = 0.001). For the negative professional identity subscale, there were main effects of both program level (graduate > undergraduate, F(1,298) = 22.120, p < 0.001) and specialization (general > HPP, F(1,298) = 8.668, p = 0.003). There was a main effect of enrollment in a HPP on the roles & responsibilities subscale (HPP > general, F(1,298) = 40.111, p < 0.001) (Fig. 3). No main effects were found for age and gender (p > 0.05).

### Q-methodology survey results

From the 71 respondents, 54 participants loaded on three factors representing three major viewpoints of students. Based on the Q-statements that loaded on each factor and on students’ qualitative feedback, the authors named the factors as “Factor 1: It’s about the team”, “Factor 2: It’s about the patient” and “Factor 3: It’s about me”. Participant rotated (orthogonal varimax) factor loadings and statements scores by factor are in online supplements 3 and 4, respectively. Statements loading in each factor and students’ representative quotes are in Table 3.

Factor 1: “It’s about the team” had 24 participants loading on it. This group was mainly composed of students from undergraduate and general programs (n = 11; 46%). Participants in this group were generally very oriented for IPE and teamwork and not specifically for healthcare purposes, as indicated by the RIPLS sentences they highly agreed with (i.e., rated + 3), such as “For small-group learning to work, students need to trust and respect each other” ; and sentences they highly disagreed with (i.e., rated − 3), such as “It is not necessary for undergraduate health care students to learn together”. When justifying their ratings, participants further highlighted the need for team work not only in healthcare but in all aspects of working life: “I think group work is beneficial for so many reasons, not just if you want to go into the healthcare field. You need it in almost every field out there in the world and you’re going to be working with people everywhere, so it’s always good to be ready for that.”

Nineteen participants loaded in factor 2 “It’s about the patient”. Participants in this group were mainly students from graduate health programs (n = 15; 79%) who valued IPE with a goal of enhancing the care provided to the patient. This perspective is well represented by the following sentences with which they agreed (i.e., rated 2) or highly agreed (rated 3) with: “Patients would ultimately benefit if health care students worked together to solve patient problems”; and “Shared learning with other health care students will increase my ability to understand clinical problems”. Participants’ quotes also recognised the limited benefits of healthcare professionals working isolated in the rehabilitation process as these patients often present multiple and complex health problems:

In any health profession, the ultimate goal is to help a patient reach their rehabilitation goals. Each profession has a limited scope of practice, yet the patient may require a variety of treatments to reach their goals. By understanding what other professions are contributing to a patient or client’s rehabilitation, professionals as a group can decide what treatment options are best and how they will work well with one another.

Eleven participants loaded in factor 3 (“It’s about me”). This group was mainly composed of students from undergraduate health programs (n = 6; 55%) who believed their learning needs and requirements were greater than those of students from other programs and under appreciated the value of IPE. This belief is reflected in following sentences they agreed and strongly disagreed with, respectively: “I have to acquire much more knowledge and skills than other health care students”; and “Shared learning with other health care students will help me to communicate better with patients and other professional”.

Finally, there were six consensus statements where all groups were neutral or in slight agreement with the notion that shared learning with will improve relationships and teamwork after graduation and they all disagreed that “learning with other healthcare students was a waste of time” and that “the function of most allied health professions is mainly to provide support for doctors” (online supplement 4).

**Table 3:** Factors identified, differentiating statements and qualitative justification from participants

	RIPLS Statement	Example Qualitative Justification
Factor 1: It’s about the team (n = 24)		
Strongly Agree	For small group learning to work, students need to trust and respect each other	*I think it is really important to work with other health care students to understand and get to know other student’s perspectives and their roles.*
Strongly Disagree	It is not necessary for undergraduate health care students to learn together	*It is imperative that health care students learn how to work with others, and that should start with fellow students.*
Factor 2: It’s about the patient (n = 19)		
Strongly Agree	Patients would ultimately benefit if health care students worked together to solve patient problems	*Everyone brings a different knowledge set to the table and a combination of this knowledge can lead to the best outcome for the patient.*
Strongly Disagree	I am not sure what my professional role will be	*Although still a student, I do know that my ultimate professional role in the end will be an occupational therapist, and I will be able to assist individuals in overcoming challenges and begin or continue engaging in meaningful daily activities, while still working in a interprofessional team to understand clients as a whole.*
Factor 3: It’s about me (n = 11)		
Strongly Agree	Learning with other students will make me a more effective member of a health care team	*It is because I need to learn more knowledge so that if other health care students does not how to apply that knowledge on a patient then I can help that individual.*
Strongly Disagree	Shared learning with other health care students will help me to communicate better with patients and other professionals	-*

**Legend:** * the overall score for this statement was the lowest for factor 3 (z score − 1.7) and it was re-assigned a -3 as part of the ranking, but no comment is available

**Fig. 4:**
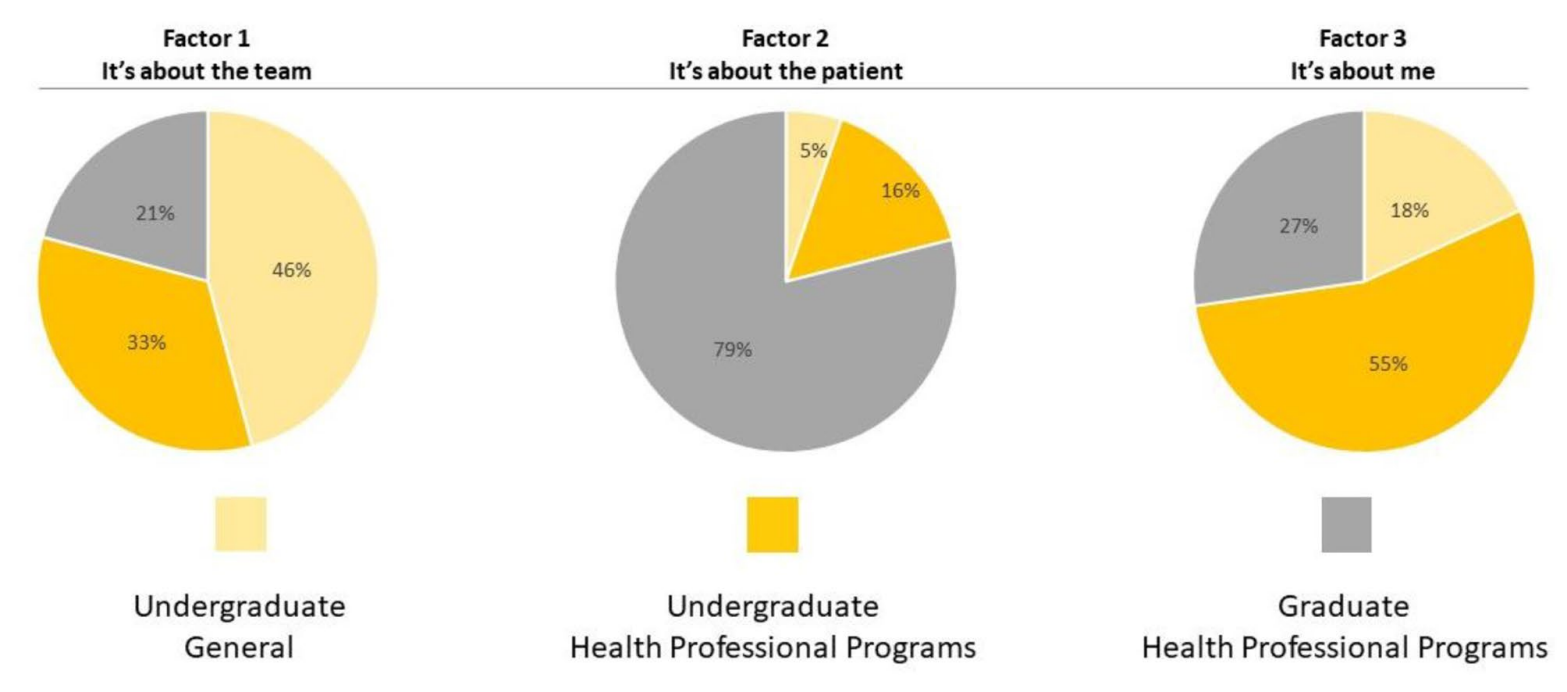
Proportion of participants from each level/specialization group loading onto each factor

The distribution of program specialisation was statistically significant between the factors (p = 0.04) as demonstrated in Fig. 4. Specifically, Factors 1 and 2 had a greater proportion of undergraduate students, while factor 2 was composed primarily of graduate students.

## Discussion

This manuscript employed the novel method of combining both Likert scale and Q-methodology surveys to better understand the readiness for IPE of first year health science students in terms of their level of study and program specialization. The RIPLS Likert results demonstrated that overall, first year students in health science programs have high levels of readiness for IPE, with students from graduate and HPP demonstrating the highest scores. The Q-methodology survey identified which components of IPE students valued the most and further described how these preferences vary across program level and specialization. Together, this complementary information will allow programs to re-evaluate and re-shape the IPE opportunities offered to first year students to ensure better alignment with their needs and increase their readiness for IPE and interprofessional collaboration.

Similar to previous studies, all students who responded showed high readiness for IPE (average RIPLS scores from 3.04 to 4.51 out of 5) [28–32], with the highest scores achieved by students from graduate and HPP. These differences are particularly noted in the negative professional identity subscale on which both graduate and specialized programs scored the highest. This subscale item suggests students entering a graduate or specialized program may have a negative or unclear view regarding their eventual professional role and responsibility. While this may be due to the lack of experience and exposure to an interprofessional and/or healthcare environment [15, 24] it informs institutions about the need to include a focused explanation of each profession’s role and responsibilities in healthcare institutions within their curriculum.

Contrary to previous studies demonstrating an effect of age and gender [28, 31, 33], results of the current study found no such effects. Compared with previous literature, our study presented a more balanced distribution of men and women and a smaller age range, which may explain these differences. Similar results were found by Lestari, Yuliyanti [34], whom presented a similar gender distribution as in our study.

The RIPLS has been one of the most recommended instruments to assess readiness for IPE as it has undergone large patterns of validity and reliability testing with different samples [35]. However, recent studies report on the skewness and the existence of a strong ceiling effect of the RIPLS [36], which may impair its discrimination ability. Although not affecting the total score, a ceiling effect was observed in our study in the positive professional identity subscale, with 17% of the students achieving the highest score possible in the scale [37]. Additionally, more than 70% of the participants achieved a mean score of 4 or more (out of 5) in the total score, indicating a clear shift in the results to the right (e.g., more positive scores favouring IPE parameters), and limiting our ability to identify these areas of IPE valued, more or less, by students – a limitation we were able to address via the use of Q-methodology in the adjacent sample.

Through using the Q-methodology survey we were able to show students with different characteristics value different components and outcomes of IPE experiences and differentially prioritize the value of teamwork (group 1), patient care (group 2) and self-development (group 3). It was interesting to observe a higher proportion of undergraduates, and indeed those in general programs, were focused on how IPE would potentially benefit their skills, whilst more experienced students focused on the benefits to the patient. Considering the ultimate goal of IPE is to improve quality of the care provided to the patient [2], it seems these results align with the RIPLS scores on the Likert scale, which showed graduate students are overall more well prepared for IPE, and previous literature which has shown former experience plays a role in attitudes toward interprofessional practice [38].Further, it suggests that students entering graduate-level specialized programs already see the necessary value and emphasis on patient care associated with their program.

The identification of different needs and values of IPE of the first-year students in health sciences programs has several practical implications in selecting the most appropriate key elements for IPE according to each student group’s needs. For example, students who are focused on self development (primarily undergraduate), may benefit from group reflective exercises, within safe learning environments, to develop an appreciation and understanding of each other’s roles, their unique backgrounds and the distinctive and complementary professional perspectives on clinical decision making [39]. Alternatively, more experienced students who display a higher IPE readiness and who were focused on the benefit to the patients (primarily graduate), may benefit from real or simulated experiences with models of collaborative practices in both hospital and community health settings [39].

### Strengths and limitations

To the authors’ knowledge, this is the first study to demonstrate the benefits of combining two different methods, namely Likert scales and Q-methodology to explore the readiness for IPE of first year students in health science programs. Additionally, given the limited postgraduate studies in this area [29, 40], this study expands on the knowledge base for postgraduate-level practitioners as well as the inclusion of health professional and general students to the sample population.

A potential limitation with the current study is that, although the participant pool included a wide variety of health science programs, the data was collected from only one university, and the results may not be generalizable to students at other institutions. It is also worth noting that with Q-methodology generalizations rarely occur beyond the immediate set of participants [27]. The nature of the study design may have also affected the study outcome due to the possibility of selection bias (i.e., students interested in IPE could have been more motivated to answer) and the inability to measure interpersonal confounders, such as previous degrees and professional activities. Future studies should include more gender options, as more than 50% of students who participated in this study identified with one gender or preferred to not report gender. An increase in the available gender options may increase in the comprehensiveness and representativeness of the results.

## Conclusion

This study harnessed the numeric and generalizable strengths of Likert scales, alongside the subjective and psychologically grounded perspectives emerging from Q-methodology, to characterize first year health science students’ readiness for IPE and their unique understanding around the benefits of IPE. Overall, students demonstrated high levels of IPE readiness upon entry to their program, with graduate students’ readiness exceeding that of their undergraduate counterparts. Further, the needs and values of students shift from being self-focussed to patient centered as they progress from undergraduate to graduate level, and general to specialized programs. Together, this information both underscores the need to develop and administer targeted IPE initiatives to support varying levels of students throughout their academic programs, but also provides actionable insight into which strategies and targets may be most successful and for whom.

### Electronic supplementary material

Below is the link to the electronic supplementary material.

Supplementary Material 1

## Data Availability

The datasets used and analysed during the current study are available from the corresponding author on reasonable request.

## Abbreviations

HPP: health professional programs
IPE: interprofessional education
RIPLS: interprofessional learning Likert scale
SPSS: Statistical Package for the Social Sciences
ANCOVA: two-way analysis of covariance
