# Supplementary material for: Readiness for interprofessional learning among health science students: a cross-sectional Q-methodology and likert-based study
Source: BMC Med Educ. 2023 Aug 18;23:583. doi: 10.1186/s12909-023-04566-w (PMC10439564; doi:10.1186/s12909-023-04566-w)
Supplement: Supplementary file 1 — Supplementary Material 1 [file 12909_2023_4566_MOESM1_ESM.docx]

**ONLINE SUPPLEMENTS**

**Readiness for Interprofessional Learning Among Health Science Students:**

**A cross-sectional Q-methodology and Likert-Based study**

Ana Oliveira^a-d^, Danielle Brewer-Deluce^a,e^, Noori Akhtar-Danesh^e^, Sarah Wojkowski^a^

^a^Program for Interprofessional Practice, Education and Research (PIPER), McMaster University, Hamilton, Canada; ^b^Respiratory Medicine, West Park Healthcare Centre, Toronto, Canada; ^c^Lab3R – Respiratory Research and Rehabilitation Laboratory, School of Health Sciences, University of Aveiro (ESSUA), Aveiro, Portugal; ^d^iBiMED – Institute of Biomedicine, Department of Medical Sciences, University of Aveiro, Aveiro, Portugal; ^e^Faculty of Health Sciences, McMaster University, Hamilton, Canada

**Corresponding author**: Ana Oliveira, 1400 Main Street West, Hamilton, ON, L8S 1C7, 647-765-1525, araujoda@mcmaster.ca

Online supplement 1: Readiness for interprofessional learning scale.

| 1 | Learning with other students will make me a more effective member of a health care team |
| --- | --- |
| 2 | Patients would ultimately benefit if health care students worked together to solve patient problems |
| 3 | Shared learning with other health care students will increase my ability to understand clinical problems |
| 4 | Learning with other health care students before qualification would improve relationships after qualification |
| 5 | Communication skills should be learned with other health care students |
| 6 | Shared learning will help me to think positively about other professionals |
| 7 | For small-group learning to work, students need to trust and respect each other |
| 8 | Team-work sills are essential for all health care students to learn |
| 9 | Shared learning will help me to understand my own limitations |
| 10 | I don’t want to waste time learning with other health care students |
| 11 | It is not necessary for undergraduate health care students to learn together |
| 12 | Clinical problem solving can only be learned with students from my own discipline |
| 13 | Shared learning with other health care students will help me to communicate better with patients and other professionals |
| 14 | I would welcome the opportunity to work on small group projects with other health care students |
| 15 | Shared learning will help to clarify the nature of patient or client problems |
| 16 | Shared learning before qualification will help me become a better team player |
| 17 | I am not sure what my professional role will be |
| 18 | I have to acquire much more knowledge and skills than other health care students outside of my discipline |
| 19 | The function of most allied health professions is mainly to provide support for doctors |


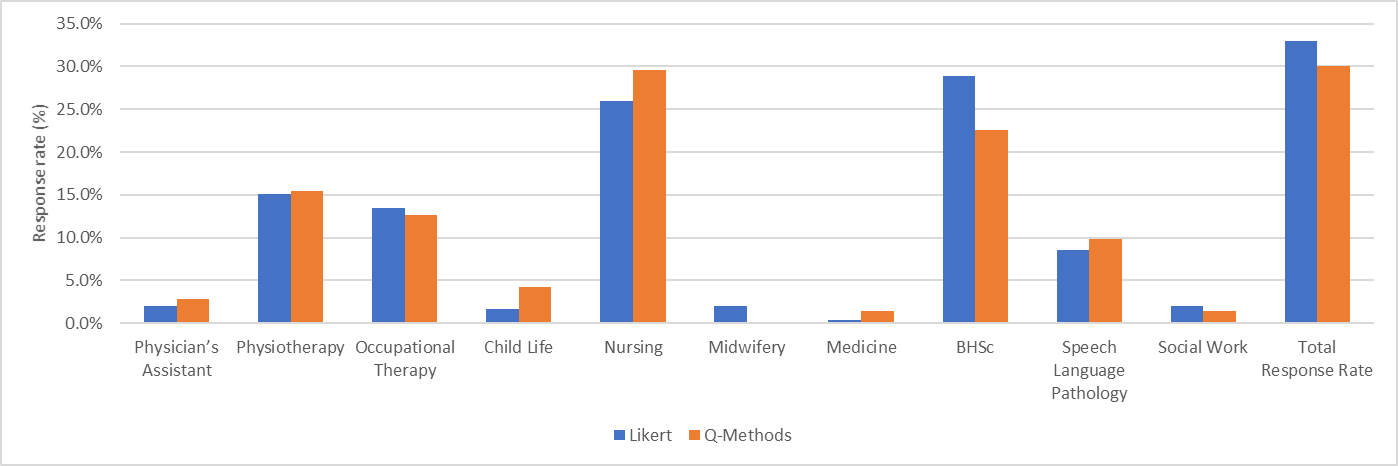
 Online supplement 2. Response rate for Likert scale or the Q-methods RIPLS per program.

Online supplement 3: Participant Rotated (Orthogonal Varimax) Factor Loadings

| Participant | Factor 1 | Factor 2 | Factor 3 |
| --- | --- | --- | --- |
| 6 | 0.54 ^a^ |  |  |
| 9 | 0.57 ^a^ |  |  |
| 22 | 0.70 ^a^ |  |  |
| 24 | 0.75 ^a^ |  |  |
| 26 | 0.69 ^a^ |  |  |
| 31 | 0.56 ^a^ |  |  |
| 34 | 0.76 ^a^ |  |  |
| 36 | 0.53 ^a^ |  |  |
| 42 | 0.55 ^a^ |  |  |
| 44 | 0.63 ^a^ |  |  |
| 48 | 0.76 ^a^ |  |  |
| 49 | 0.64 ^a^ |  |  |
| 51 | 0.69 ^a^ |  |  |
| 55 | 0.76 ^a^ |  |  |
| 58 | 0.64 ^a^ |  |  |
| 59 | 0.76 ^a^ |  |  |
| 60 | 0.69 ^a^ |  |  |
| 62 | 0.77 ^a^ |  |  |
| 63 | 0.65 ^a^ |  |  |
| 68 | 0.72 ^a^ |  |  |
| 73 | 0.74 ^a^ |  |  |
| 75 | 0.63 ^a^ |  |  |
| 76 | 0.74 ^a^ |  |  |
| 78 | 0.58 ^a^ |  |  |
| 3 |  | 0.83 ^a^ |  |
| 8 |  | 0.63 ^a^ |  |
| 10 |  | 0.65 ^a^ |  |
| 11 |  | 0.85 ^a^ |  |
| 12 |  | 0.61 ^a^ |  |
| 13 |  | 0.65 ^a^ |  |
| 15 |  | 0.87 ^a^ |  |
| 19 |  | 0.76 ^a^ |  |
| 20 |  | 0.64 ^a^ |  |
| 25 |  | 0.89 ^a^ |  |
| 27 |  | 0.63 ^a^ |  |
| 32 |  | 0.65 ^a^ |  |
| 35 |  | 0.50 ^a^ |  |
| 41 |  | 0.69 ^a^ |  |
| 47 |  | 0.71 ^a^ |  |
| 56 |  | 0.57 ^a^ |  |
| 57 |  | 0.56 ^a^ |  |
| 61 |  | 0.63 ^a^ |  |
| 69 |  | 0.67 ^a^ |  |
| 16 |  |  | 0.55 ^a^ |
| 18 |  |  | 0.65 ^a^ |
| 23 |  |  | 0.53 ^a^ |
| 28 |  |  | 0.70 ^a^ |
| 29 |  |  | 0.62 ^a^ |
| 37 |  |  | 0.47 ^a^ |
| 39 |  |  | 0.64 ^a^ |
| 53 |  |  | 0.63 ^a^ |
| 65 |  |  | 0.74 ^a^ |
| 70 |  |  | 0.62 ^a^ |
| 74 |  |  | 0.62 ^a^ |
| ** indicates* *factor upon which each participant significantly loaded (p ≤ 0.05)* | | | |

Online supplement 4 - RIPLS Statements with scores grouped by factor

| RIPLS Statement | Factor^a^ | | |
| --- | --- | --- | --- |
|  | 1 | 2 | 3 |
| Learning with other students will make me a more effective member of a health care team | **0** | 1 | 3 |
| Patients would ultimately benefit if health care students worked together to solve patient problems | 0 | **3** | -1 |
| Shared learning with other health care students will increase my ability to understand clinical problems | 0 | **2** | 0 |
| ^b^ Learning with other health care students before qualification would improve relationships after qualification | 1 | 0 | 0 |
| Communication skills should be learned with other health care students | 1 | **-1** | 0 |
| Shared learning will help me to think positively about other professionals | -1 | 1 | 1 |
| For small-group learning to work, students need to trust and respect each other | **3** | **-2** | **2** |
| Team-work sills are essential for all health care students to learn | 2 | **0** | 1 |
| ^b^ Shared learning will help me to understand my own limitations | 0 | 1 | 1 |
| ^b^ I don’t want to waste time learning with other health care students | -2 | -1 | -1 |
| It is not necessary for undergraduate health care students to learn together | **-3** | 0 | -1 |
| Clinical problem solving can only be learned with students from my own discipline | -1 | -1 | -2 |
| Shared learning with other health care students will help me to communicate better with patients and other professionals | 2 | 1 | **-3** |
| ^b^ I would welcome the opportunity to work on small group projects with other health care students | 0 | 0 | 0 |
| Shared learning will help to clarify the nature of patient or client problems | **-1** | **2** | **1** |
| ^b^ Shared learning before qualification will help me become a better team player | 1 | 0 | 0 |
| I am not sure what my professional role will be | **1** | -3 | -1 |
| I have to acquire much more knowledge and skills than other health care students outside of my discipline | -2 | -2 | **2** |
| ^b^ The function of most allied health professions is mainly to provide support for doctors | -1 | -1 | -2 |

**Legend:** ^a^Factors: Factor 1: It’s about the team”, “Factor 2: It’s about the patient” and “Factor 3: It’s about me”; ^b^Consensus statements (no differences between factors); Distinguishing statements by factor are in bold (indicate significantly different value from other factors, P ≤ 0.05).
